# A new family of hybrid virophages from an animal gut metagenome

**DOI:** 10.1186/s13062-015-0054-9

**Published:** 2015-04-25

**Authors:** Natalya Yutin, Vladimir V Kapitonov, Eugene V Koonin

**Affiliations:** National Center for Biotechnology Information, National Library of Medicine, National Institutes of Health, Bethesda, MD 20894 USA

## Abstract

Search of metagenomics sequence databases for homologs of virophage capsid proteins resulted in the discovery of a new family of virophages in the sheep rumen metagenome. The genomes of the rumen virophages (RVP) encode a typical virophage major capsid protein, ATPase and protease combined with a Polinton-type, protein primed family B DNA polymerase. The RVP genomes appear to be linear molecules, with terminal inverted repeats. Thus, the RVP seem to represent virophage-Polinton hybrids that are likely capable of formation of infectious virions. Virion proteins of mimiviruses were detected in the same metagenomes as the RVP suggesting that the virophages of the new family parasitize on giant viruses that infect protist inhabitants of the rumen.

This article was reviewed by Mart Krupovic and Kenneth Stedman; for complete reviews, see the Reviewers’ Reports section.

**Electronic supplementary material:**

The online version of this article (doi:10.1186/s13062-015-0054-9) contains supplementary material, which is available to authorized users.

## Findings

With the rapid increase of the quantity and quality of available metagenomics sequences, metagenomes have become a rich source for the discovery of novel viruses [1-3]. A prominent case in point is the recent discovery of a novel, abundant and diversified group of viruses that are chimeras of genes from single-stranded DNA and positive-strand RNA viruses [4-7]. So far none of these viruses has been isolated in the form of infectious particles but metagenomic sequence analysis suggests that they might be major components of viromes in various environments. Equally striking is the assembly of a novel bacteriophage that apparently accounts for a substantial part of the human gut virome but remained unknown until the advent of metagenomics [8]. These success stories prompt focused mining of metagenomic databases for novel genetic elements.

We were interested in mining metagenomes to explore the diversity of virophages, an unusual group of viruses that parasitize on giant viruses of the family Mimiviridae. The known virophages possess double-stranded, typically circular DNA genomes of about 20 kilobase pairs (kbp) and icosahedral virions comprised of highly derived double jelly-roll capsid proteins [9,10]. So far 3 virophages have been isolated as infectious particles and shown to be associated with different members of the family Mimiviridae [11-13], and 9 additional virophage genomes have been assembled from aquatic metagenomes [14-16]. The first virophage, named Sputnik, was discovered as a parasite of Acanthamoeba castellani mimivirus [11], and reproduction of the related Zamilon virophage is supported by several mimiviruses [13]. The third identified virophage, denoted Mavirus, is a parasite of the giant virus that infects the flagellate *Cafeteria roenbergensis* [12]. Sputnik and Mavirus are prototypes of two distinct families of virophages; the founding member of a third family is Organic Lake Virophage (OLV) whose genome has been assembled from a metagenome and that is thought to parasitize on Organic Lake phycodnaviruses [14] that subsequently have been reclassified as putative members of the extended family Mimiviridae [17,18]. So far all (putative) virophage genomes assembled from metagenomes fall into one of the above three families [19].

The genomes of Mavirus [12] and the related Ace Lake Mavirus (ALM) virophage [15] contain two genes that are missing in other virophages, namely a protein-primed family B DNA polymerase (pPolB) and an integrase, both of which are most closely related to the respective homologous genes of a vast family of large eukaryotic self-synthesizing DNA transposons known as Polintons (Mavericks) [20,21]. The sharing of these two genes between Mavirus and Polintons along with the conservation of genes encoding predicted protease and ATPase in all Polintons suggested an evolutionary relationship between these two classes of selfish genetic elements [12,19]. Further analysis of the Polinton genomes buttressed this hypothesis by showing that most of these transposons actually encode homologs of the major and minor capsid proteins of icosahedral viruses [22]. Thus, Polintons are most likely capable of forming virions and lead a dual lifestyle that combines features of transposons and viruses. These findings have prompted a broad-scale evolutionary scenario according to which the Polintons (Polintoviruses) were the ancestors of the virophages and several other groups of dsDNA viruses, including the large and giant viruses of the proposed order “Megavirales”, and dsDNA plasmids of eukaryotes [23]. Under this scenario, the Polintons comprise a pool of diverse genetic elements, and various other eukaryotic parasitic elements, in particular adenoviruses, virophages, and fungal cytoplasmic plasmids, evolved from different groups of polintons [23].

We sought to harness the potential of metagenomics for the discovery of substantially novel virophages, distinct from the Sputnik, Mavirus and OLV families. All sequenced virophage genomes encompass a conserved gene module that encodes proteins involved in virion morphognesis, namely major and minor capsid proteins, packaging ATPase and capsid maturation protease, but otherwise, possess highly variably gene repertoires [19]. Among the four conserved proteins of the virophages, the protease and the ATPase share significant sequence similarity with homologs from diverse viruses whereas the sequence of the minor capsid protein is poorly conserved even among the virophages. Thus, the best probe for discovery of putative new virophages is the major capsid protein (MCP) which adopts a derived double jelly-roll fold [10] and is well-conserved in the virophages but shows no significant similarity to any non-virophage proteins [19].

We used four MCP sequences from diverse virophages to search the metagenomic nucleotide databases that are available via GenBank and detected multiple hits (E-value < 10). The respective metagenomic sequences were translated and used as queries for BLASTP to search the non-redundant protein sequence database, in order to further ascertain their provenance. As a result of this search, 35 Open Reading Frames (ORFs) and ORF fragments from 6 metagenomics databases, namely, Activated sludge metagenome (AS), Bioreactor metagenome (BR), Freshwater sediment metagenome (FWS), Gut metagenome (Gut), Marine metagenome (Mar), and Wastewater metagenome (WW), were identified as likely virophage MCPs (see Additional file 1).

**Figure 1:**
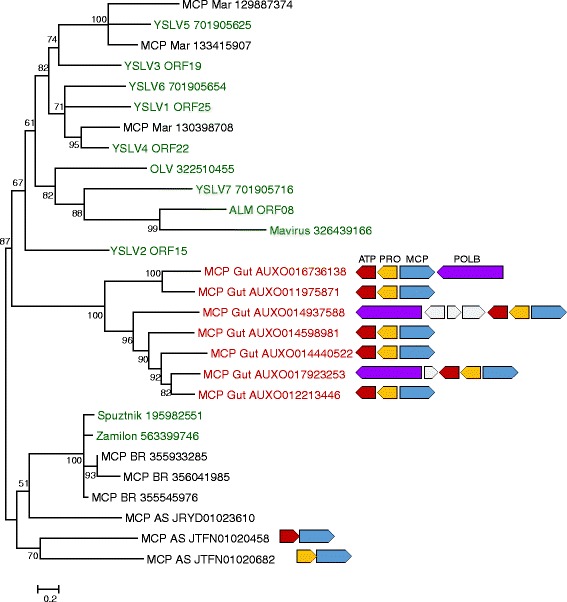
Phylogenetic tree of the major capsid proteins of virophages. The inferred gene organizations in the RVP genomes are shown at the respective branches. The genes are shown as block arrows, with the orientation of the MCP gene chosen as the reference. Green, virophages; red, rumen metagenome sequences. The numbers at the internal branches represent ELW values from 1,000 local rearrangements given as percentage points. Branches with bootstrap support less than 50% are shown as multifurcations. Metagenomic sequence names include the abbreviation for the respective metagenome and nucleotide sequence ID. The metagenomes were as follows: Activated sludge metagenome (AS), Bioreactor metagenome (BR), Gut metagenome (Gut), Marine metagenome (Mar). Virophage abbreviations: ALM, Ace Lake Mavirus; OLV, Organic Lake virophage; YSLV, Yellowstone Lake Virophage. Gene abbreviations: ATP, ATPase; PRO, cysteine protease; MCP, major capsid protein; POLB, DNA polymerase. For virophages present in Genbank, Genbank protein IDs follow virophage names.

Sixteen metagenomic MCP sequences were aligned with the MCP sequences from complete virophage genomes, and the resulting multiple alignment was used for phylogenetic tree construction. In the resulting tree, the putative virophage proteins from the sludge and bioreactor metagenomes clustered within the Sputnik-like virophage family whereas those from the marine metagenome fell within the OLV-like family (Figure 1). In contrast, the putative virophage MCPs from the gut metagenome (more specifically, all these sequences originate from the sheep rumen, so hereinafter we refer to this sequence collection as the rumen metagenome) formed a distinct, strongly supported group (Figure 1). We further examined the metagenomic sequence contigs that encompassed the putative virophage MCP genes (see Additional file 1). The contigs from the sludge, marine and bioreactor metagenomes were short and, with a single exception, did not contain additional genes. In contrast, the rumen metagenome contigs were longer, up to 26,209 basepairs. The ORFs present in these contigs were translated and searched against the non-redundant protein sequence database at the NCBI, resulting in the identification of a typical virophage morphogenetic module (Figure 1).

**Figure 2:**
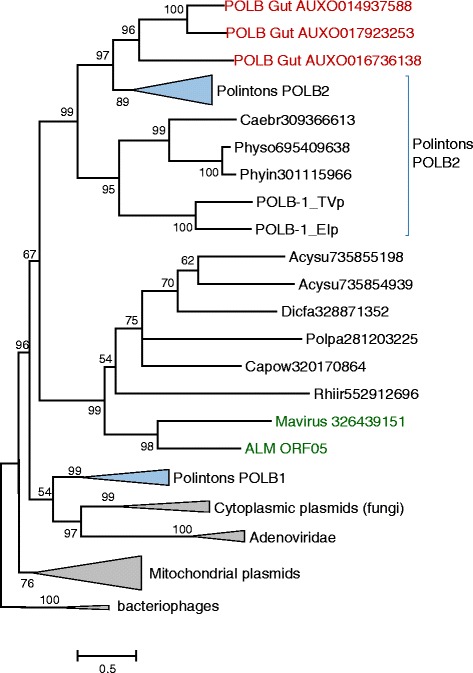
Phylogenetic tree of the protein-primed DNA polymerases. The designations are as in Figure 1. Branches in several clades are collapsed and shown as triangles. Eukaryotic species abbreviations: Acysu, *Acytostelium subglobosum*; Caebr, *Caenorhabditis briggsae*; Capow, *Capsaspora owczarzaki*; Dicfa, *Dictyostelium fasciculatum*; Phyin, *Phytophthora infestans*; Physo, *Phytophthora sojae*; Polpa, *Polysphondylium pallidum*; Rhiir, *Rhizophagus irregularis*; EI *Entamoeba invadens*; TV, *Trichomonas vaginalis*.

Phylogenetic analysis of the putative virophage ATPases and proteases supported the monophyly of the “rumen” group and its association with other virophages (see Additional file 2). The three longest contigs from the rumen metagenome encoded an additional putative protein that in database searches showed the highest similarity to pPolBs from diverse Polintons. Phylogenetic analysis of these putative pPolBs confidently placed them into the clade known as Polinton family 2, without any indication of clustering with the Mavirus pPolB (Figure 2). Thus, the results of phylogenetic analysis reported here are compatible with the previously described evolutionary trend whereby different groups of eukaryotic selfish elements evolved from within the diversity of the Polintons [23].

The remaining ORFs in the putative rumen virophage (RVP) genomes encoded proteins without significant similarities in the current databases, with the sole exception of a putative polynucleotide kinase encoded in one of the contigs (see Additional file 1).

We attempted to extend the assembly of the rumen metagenome contigs to further characterize the putative novel virophages. Identification of several overlapping contigs and terminal inverted repeats (TIRs) allowed us to assemble 3 long virophage genomes (see Additional file 3). Two of these assemblies ended in TIRs suggesting the possibility that they corresponded to complete genomes.

Thus, metagenome search described here led to the identification of a putative novel group of viriophages with several distinctive features of genome composition and architecture. The putative virophages of this RVP group, combine a morphogenetic module that is clearly related to that of virophages, with a pPolB of apparent Polinton origin. Thus, these entities seem to be virophage- Polinton hybrids. However, it appears that the RVP have to be classified as virophages rather than Polintons given that they encode a highly derived MCP that differentiates the virophages from all other viruses. All known virophages and most of the Polintons, in addition to the MCP, encode also a minor capsid protein (penton); typically, the two capsid proteins are encoded in adjacent genes [19,22]. A focused search of all RVP-encoded protein sequences for potential homologs of the minor capsid protein failed to detect any significant sequence similarity. Moreover, the genome organization of the RVP appears to leave no room for a penton gene next to the MCP gene (Figure 1). Thus, the RVP appear to lack the penton, with the implication that the structures of their putative capsids are substantially different from those of the known virophages.

The presence of the idiosyncratic structural variant of the MCP that is unique to virophages implies that the RVPs are parasites of giant viruses in the family Mimiviridae. To ascertain the presence of mimivirus sequences in the analyzed metagenomes, we searched the metagenomic libraries, in which the virophage MCPs were identified, using as queries representative sequences of mimivirus MCPs. Sequences of apparent mimivirus origin were identified in all metagenomes except for WW but were particularly numerous in the gut metagenome (see Additional file 1). Thus, all the results of metagenome analysis reported here are compatible with the conclusion that a distinct family of virophages parasitizes on mimiviruses that most likely infect protists present in the rumen; indeed, protists are common in this habitat [24,25].

The genomes of the RVPs appear to have evolved via recombination between a virophage and a Polinton. A similar evolutionary scenario seems to have produced the Mavirus which inherited from the Polinton ancestor not only the PolB but also the integrase [12]. However, phylogenetic analysis of both the virophage-derived morphogenetic module and the Polinton-derived PolB indicate that the RVP are not directly related to the Mavirus (Figure 2). Thus, virophage-Polinton chimeras have independently evolved on at least two but probably many occasions. The definitive characterization of the genomic architecture of the RVP should await complete genome analysis. Nevertheless, the presence of TIRs at the ends of the RVP contigs suggests that they have linear genomes unlike the other virophages but similar to Polintons [20]. In this respect, the RVPs resemble also an unusual putative virophage that has been discovered in association with *Phaeocystis globosa* virus (PGV), a distant relative of the mimiviruses [18]. However, there is otherwise no indication of an evolutionary affinity between the RVP and the PGV virophage (which encodes a polinton-like MCP and a primase-helicase rather than pPolB), once again indicative of parallel evolutionary trajectories. In all four RVPs with identified TIRs, the repeats are located at the very ends of contigs and cannot be extended further using the available sequence data. Together with the lack of detectable integrases in the RVP, these observations imply that the RVPs parasitize on the giant viruses without integrating into the viral genome. However, occasional integration mediated by an in-trans integrase cannot be ruled out.

## Conclusions

The identification of a new family of putative virophages with unusual features of genomic composition and architecture emphasizes the potential of metagenomes for discovery of novel classes of mobile elements. The RVPs appear to be virophage-Polinton chimeras and in this respect resemble the chimeric ssDNA viruses that have been recently discovered, also by metagenome mining [4,5,7,26]. These findings further highlight recombination and module shuffling as the central theme in the evolution of viruses and other selfish genetic elements [27]. More specifically, these results indicate that the evolutionary trajectories of Polintons (polintoviruses) and virophages have repeatedly crossed beyond the original, ancestral relationship. Conceivably, the recombination that gave rise to the RVPs involved a virophage and a viral form of a Polinton [23] (polintovirus) within a giant virus-infected amoeba which is known as a “melting pot” for the evolution of diverse selfish elements [28].

## Methods

Four virophage major capsid proteins, Mavirus GI:326439166; Sputnik virophage GI:193245560, Organic Lake virophage GI:322510455, and Yellowstone Lake virophage 7 GI:701905716, were used as queries for translating blast searches (Tblastn [29]) against whole-genome shotgun contigs from the metagenomes available at the NCBI (taxid:408169). The hits from the four searches were combined, translated using GeneMark [30] and searched against the NCBI nr database using Blastp [29].

Similarly, four mimiviridae major capsid proteins, Acanthamoeba polyphaga mimivirus GI:311977828, Cafeteria roenbergensis virus BV-PW1 GI:310831332, Acanthamoeba polyphaga mimivirus GI:311977809, and Organic Lake phycodnavirus 1 GI:322510624, were used as queries for Tblastn searches against the AS, BR, FWS, Gut, and WW metagenomes.

Protein sequences were aligned using MUSCLE [31], and gapped columns (more than 30% of gaps) and columns with low information content were removed from the alignment [32]. A preliminary tree was constructed using the FastTree program with default parameters (JTT evolutionary model, discrete gamma model with 20 rate categories) [33]. The best-fit substitution model was identified using ProtTest [34]. The final maximum likelihood tree was calculated using TreeFinder [35], with the substitution model found to be the best for a given alignment. The following substitution models were identified by ProtTest as the best fit for individual genes for which phylogenetic analysis is reported: MCP - RtREV + G + F; ATPase - LG + G + F; protease - LG + G + F; pPolB - LG + G + F. The bootstrap values represent Expected-Likelihood Weights (ELW) of 1,000 local rearrangements.

Inverted repeats were identified using Censor software [36].

## Reviewers’ reports

### Reviewer 1

Mart Krupovic, Institut Pasteur

In this Discovery Note, Yutin et al. describe a new group of putative virophages, which they discovered in the sheep rumen metagenome. These new elements differ considerably from the previously described virophages and appear to contain a chimeric genome. The virion morphogenesis module is similar to that shared by all virophages, whereas the family B DNA polymerase is more closely related to the corresponding proteins of large, virus-like DNA transposons of the Polinton/Maverick superfamily. The authors succeeded to assemble several nearly complete genomes of these novel virophages. The virophage-containing metagenome was positive for the presence of mimiviruses, as judged by the occurrence of reads coding for the mimivirus major capsid protein. This result is consistent with the conclusion that the newly identified virophages parasitize on the giant viruses. The manuscript is very clearly and logically written.

Using the major capsid protein sequences presented in Additional file 1, I could confirm the relationship between the capsid proteins of the rumen virophages with those of the previously described virophages. However, I could not access the genomic contigs of the gut metagenome. It would be desirable to present them as archived GenBank files in the supplementary information.

The morphogenetic module of virophages and polintons includes a minor capsid protein. Do the rumen virophages encode a homolog of this protein? This has to be stated in the text.

Authors’ response: *This is an important point that is explicitly addressed in the revised manuscript. It seems that the RVP genuinely lack the minor capsid protein*.

Minor comments:

The second half of the third sentence might be an overstatement.

Authors’ response: *Yes, the second reviewer also made a similar comment. We modified the sentence to indicate that the chimeric viruses “might” be major components of viromes*.

“of two distinct family” change to “families”.

Authors’ response: *corrected*

“adopts a derived jelly-roll fold” change to ?adopts a derived double jelly-roll fold”.

Authors’ response: modified as suggested.

In the last paragraph of the Findings (before conclusions), it might be worth mentioning that although Mavirus genome has been reported as a circular molecule, it contains abutted 50 bp inverted repeats (separated by 15 bp) (ref 12 in the current reference list), which could correspond to terminal inverted repeats found in polintons, PGV and RVPs.

Authors’ response: Considering the Biology Direct format, we believe that it is sufficient to include this detail here. We appreciate the reviewer pointing it out.

Labels of some of the ORFs in Additional file 3 are flipped.

Authors’ response: Corrected.

### Reviewer 2

Kenneth Stedman, Portland State University

Yutin et al., in their “Discovery Note” have performed highly sensitive searches of available metagenomic sequence datasets for sequences similar to major capsid protein genes (MCP) of known virophages. Then they used these sequences as anchors to acquire associated sequence data from the metagenomic datasets. Thereby the authors discover a new group of sequences, whose putative MCPs are similar to virophage MCPs but consist of a phylogenetically well-supported separate clade. Interestingly, some of these MCPs are associated with putative protein-primed DNA polymerase genes that are similar to some Polinton DNA polymerase genes. The authors propose a new family of virophages based on this analysis.

It is unfortunate that this manuscript is constrained by the discovery note format, as I find the Additional figures to be integral to interpretation of the results presented in the manuscript. Specifically, I find the phylogenetic trees for the putative viral ATPase and Protease genes to be at least as convincing as the phylogenetic tree presented in Figure 1. The genome maps in Additional file 3 are also extremely useful to understanding the manuscript and supporting the hypotheses of the authors.

Authors’ response: *We appreciate these thoughts but maintain that the Discovery Note format is most appropriate for these findings. The principal reason is that we do not have confirmed, complete genome sequences of the RVP and did not have an opportunity to perform any confirmatory sequencing as pointed out by the reviewer below. Therefore the short communication format appears to be the correct choice. We do agree that the trees for ATPases and proteases are as convincing as the pPolB tree and might be even easier to interpret. We have chosen the polymerase for the main body of the article because overall, this gene is more common among diverse genetic elements than either of the other two. To the best of our understanding, the additional files are integral parts of the published paper and they are accessible from the article by one click. Thus, we hope and trust that there is no problem for readers in assessing the entirety of evidence presented here*.

Figure 2 with the protein primed DNA polymerase phylogenetic tree is confusing, due to the diversity of pPolBs in Polintons. I strongly suggest a broader introduction to Polintons and their diverse pPolBs. I also think this would help with interpretation of the figure.

Authors’ response: *We are not quite sure about the source of the confusion. Potentially, the problem might lie in the fact that different groups of eukaryotic viruses and other selfish elements emerge from within the polinton diversity. i.e. apparently evolved from different groups of polintons. We emphasize this trend in two places in the revised manuscript. Beyond these clarifications that, as we hope, suffice to avoid any confusion, there does not seem to be room in this brief manuscript to introduce the polintons in greater detail*.

I, and I think most Biology Direct readers, would like more detail on some of the methods, and some clarification of some of the statements in the manuscript. These are detailed below:

First, even though our lab was the first to recognize them, I am not sure if the chimeric ssDNA and + strand RNA viruses are “major components of viromes”. I would like to think that they are. However, since most of the viromes cited, and certainly ours, were amplified using Phi29DNA polymerase, which preferentially amplifies small ssDNA virus genomes, one cannot be sure of quantification. Perhaps this is discussed in Krupovic et al., Genome Biology and Evolution, 2015, but I was unable to access this manuscript.

Authors’ response: *this is by no account a key point in the article. We might have over-reached in the original version. The statement was softened in the revision following this comment and a similar comment of reviewer 1*.

In the second sentence of the second paragraph of “Findings”, I suggest adding “known” after “The” and before “virophages” and changing “kilobases” to “kilobasepairs”?.

Authors’ response: modified as suggested.

Third paragraph of Findings: Consider including a reference to Maverick transposons at the first mention of Polintons to clarify this for researchers who have not been able to keep up with the recent literature by the authors (and others) on Polintons.

Authors’ response: *done*

Fifth paragraph of “Findings”: The definition of “hits” needs to be made here or the exact parameters used for the searches and criteria for selection given. I did a tBLASTn search with the first MCP sequence listed (Mavirus MCP) using the metagenome taxonomic id and found “hits” with high e-values and low scores. I would also list the ids of the metagenomes found here, rather than abbreviations. I disagree that this analysis “confirmed” as likely, I would say “identified”.

Authors’ response: *We indicate in the revision that at the first stage, we used the BLAST default, i.e. E-value < 10 (no statistical significance required). “Confirmed” changed to “identified” as suggested. However, we think that here taxonomic id would unnecessarily clutter the text, abbreviations should suffice. The e-values, scores and best hit IDs, as well as the IDs of metagenomics sequences, are in the Additional file*1*.*

Sixth paragraph of “Findings”: The affinity of the Sputnik-like virophage MCPs to the sheep rumen putative virophage MCPs seems very tenuous from Figure 1. Is the value (39) listed at the branch point in the tree between these two groups correct? In my interpretation this means a different grouping 61% of the time, possibly my understanding of this ELW value is incorrect. Were all long contigs in the sheep rumen metagenome analyzed here or just the ones that contained putative virophage MCP genes? If all of the long contigs corresponded to RVPs, then they may be extremely common in the sheep rumen environment.

Authors’ response: *the interpretation of ELW is basically correct. However, the trees were redone with a different method, and based on the results included in the revised version, we do not claim an affinity with the Sputnik family anymore. We analyzed all sheep rumen metagenome contigs deposited in GenBank at the time of this manuscript submission ( BioProject PRJNA202380; 5.8 gbp; 8,786,927 contigs; AUXO000000000.1;*http://www.ncbi.nlm.nih.gov/Traces/wgs/?val=AUXO01#contigs*), and the contigs detailed in Additional file*3*are the only one encoding virophage-related proteins. Thus, RVP are not particularly common in the sheep rumen.*

Seventh paragraph: The assemblies of the metagenomic contigs shown in Additional file 3 are very interesting and quite convincing. However methods must be described as to how these contigs were assembled. It appears that they were assembled in multiple different ways.

Authors’ response: *The assemblies reported here were obtained using BLASTN searches as implemented in Censor. A detailed figure legend is included in the revised Additional file*3*. each contig encoding an MCP homolog was used as a query in a Censor search against the full set of all sheep rumen metagenome contigs (8,786,927 contigs, 5.8 Gbp). Using Censor, we have not found significant overlaps of the termini of the assembled virophages with any other contigs.*

Eighth paragraph: I disagree that the RVP group of putative virophages is “clearly related” to the Sputnik-like virophages (based on Figure 1, if Additional file 2 is added this would make the argument much stronger).

Authors’ response: *We have redone the trees and removed the noted statement. What is indeed clear, is the relationship of the morphogenetic module of the RVP with that of the virophages (in general). The RVP are a distinct, new family of virophages. The text was modified accordingly*.

Ninth paragraph: The possible relationship between the RVPs and mimivirus-like sequences in the metavirome is clearly speculative, but some mention should be made here whether these viruses are likely to be endogenous or of something consumed by the sheep. The statement “particularly numerous” is imprecise, some quantification, at least relative to the other metagenomes, should be given here.

Authors’ response: *Given that virophages so far have been shown to parasitize only giant viruses of the family Mimiviridae, we consider the relationship between the RVP and the mimiviruses that appear to be present in the same habitat, judged from metagenome analysis, to be highly plausible although, certainly, still a conjecture. The possibility that the mimiviruses come from food and do not reproduce in the rumen is difficult to fathom given the diet of sheep that is hardly rich in amoeba. “Particularly numerous” relates to the ratio of extended-Mimiviridae-like MCPs in rumen metagenome compared to other metagenomes analyzed for this matter, as shown in Additional file*1*. We do not think the numbers need to be included in the article. A particularly interested reader can calculate using Additional file*1*.*

Tenth paragraph: The analysis of TIRs should also include analysis of internal IRs, if any, in the sequences. If original metagenomic sequence reads are available, this could address whether the identified IRs are actually terminal. I would describe the PGV putative virophage in more detail, does it just have TIRs but none of the other genes? At the end of the paragraph, I believe the authors mean “in trans” instead of “in-trance”.

Authors’ response: *There were no internal IRs. A clarification on PGV virophage-encoded proteins added. The typo corrected*.

Conclusions: I completely agree with the incredible potential of metagenomes as sources of new virus genomes. It should be mentioned that, in the case of the chimeric ssDNA viruses, many of these have been reconfirmed by amplification and resequencing from original samples, which I do not believe has been done for these RVPs.

Authors’ response: *There was indeed no amplification and resequencing involved here. However, we do not believe this disclaimer belongs in the Conclusions. Suffice it to be here for the interested reader to see.*

Methods: For the nr database searches, were all of the putative MCP hits searched individually or as a consensus sequence or HMM? Please specify. How were the branch support values calculated?

Authors’ response: *Individual sequences were used as queries for database searches. Neither Tblastn nor Blastp work with profiles, so this is clear as written. The sentence regarding branch support was changed.*

“Author contributions” and “Acknowledgements” appear to be duplicated in the manuscript.

Authors’ response: *Corrected*.

Figure legends: I suggest removing “Evolutionary provenance of the rumen virophages” from both figure titles.

Authors’ response: *done, with an additional modification*.

I also suggest splitting the legend instead of putting all in Figure 1, as Figure 1 has no multifurcations or collapsed branches.

Authors’ response: *done*

A number of abbreviations are missing in this legend, such as ALM, YSLV, ATP, PRO, MCP, and pPOLB (and E8 from Figure 2).

Authors’ response: *included*

It is not clear to me that bootstrap values of less than 50% are made into multifurcations, as 18, 31, 37, and 39 are listed. If ELWs are not bootstraps, this should be made clear in the legend or text or both.

Authors’ response: *Multifurcations have been mentioned mistakenly – removed. ELWs can be interpreted as bootstrap*.

It is not clear which ORFs are meant in the YSLV sequences, I would include accession numbers for all individual sequences shown in the trees.

Authors’ response: *included*

The orientation of the cartoons for the RVPs seems random, I would orient all of them in the same way, except for those which are clearly different due to the relative orientation of the pPolB genes. I would also identify which of these contain TIRs.

Authors’ response: *Yes, these are very good suggestions. The cartoons reoriented based on the orientation of the MCP gene. The TIR are not adjacent to these conserved genes and accordingly are shown in Additional file*3.

Additional file 1: Is the annotation in this table from this study?

Authors’ response: yes, it is

Additional file 2: Include in manuscript and be very careful to define all abbreviations and list accession numbers.

Authors’ response: T*he suggestion is not entirely clear. If this is about making it a main figure instead of an Additional File, the reasons for not doing this are explained above. Abbreviations and accessions double checked and amended.*

Additional file 3: Either include ORFs in A or split up in Figure 1 (or both).

Authors’ response: *We included selected ORFs in virophage A (Additional file*3*): POLB, ATPase, cysteine protease, MCP, and one unclassified protein (“24”), other 15 unclassified proteins are not shown (they are not present in other virophages).*

## Additional files

Additional file 1:
**Annotated translation of the virophage-related contigs from metagenomes.**
Additional file 2:
**Phylogenetic trees for packaging ATPase and capsid maturation protease.**
Additional file 3:
**Nearly complete virophage genomes from the gut metagenome.**
