# Supplementary material for: A new family of hybrid virophages from an animal gut metagenome
Source: Biol Direct. 2015 Apr 25;10:19. doi: 10.1186/s13062-015-0054-9 (PMC4409740; doi:10.1186/s13062-015-0054-9)
Supplement: Additional file 2: — Phylogenetic trees for packaging ATPase and capsid maturation protease. [file 13062_2015_54_MOESM2_ESM.pptx]

## Slide 1
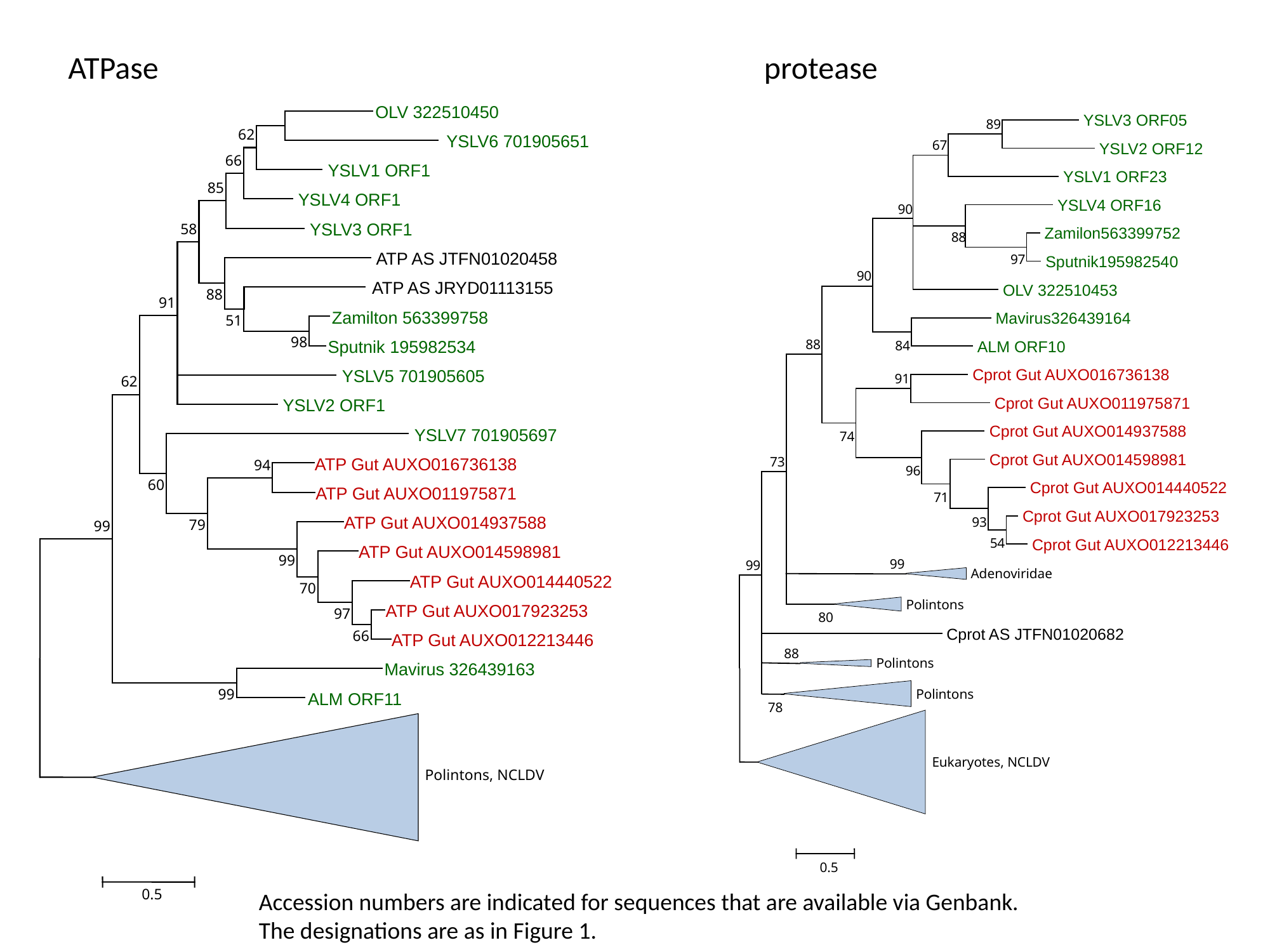

ATPase
protease
 OLV 322510450
62
 YSLV6 701905651
66
 YSLV1 ORF1
85
 YSLV4 ORF1
 YSLV3 ORF1
58
 ATP AS JTFN01020458
 ATP AS JRYD01113155
88
91
 Zamilton 563399758
51
98
 Sputnik 195982534
 YSLV5 701905605
62
 YSLV2 ORF1
 YSLV7 701905697
 ATP Gut AUXO016736138
94
60
 ATP Gut AUXO011975871
 ATP Gut AUXO014937588
79
99
 ATP Gut AUXO014598981
99
 ATP Gut AUXO014440522
70
 ATP Gut AUXO017923253
97
66
 ATP Gut AUXO012213446
 Mavirus 326439163
99
 ALM ORF11
 Polintons, NCLDV
0.5
 YSLV3 ORF05
89
67
 YSLV2 ORF12
 YSLV1 ORF23
 YSLV4 ORF16
90
 Zamilon563399752
88
97
 Sputnik195982540
90
 OLV 322510453
 Mavirus326439164
88
 ALM ORF10
84
 Cprot Gut AUXO016736138
91
 Cprot Gut AUXO011975871
 Cprot Gut AUXO014937588
74
 Cprot Gut AUXO014598981
73
96
 Cprot Gut AUXO014440522
71
 Cprot Gut AUXO017923253
93
 Cprot Gut AUXO012213446
54
99
99
 Adenoviridae
 Polintons
80
 Cprot AS JTFN01020682
88
 Polintons
 Polintons
78
 Eukaryotes, NCLDV
0.5
Accession numbers are indicated for sequences that are available via Genbank.
The designations are as in Figure 1.
