# Supplementary material for: A new family of hybrid virophages from an animal gut metagenome
Source: Biol Direct. 2015 Apr 25;10:19. doi: 10.1186/s13062-015-0054-9 (PMC4409740; doi:10.1186/s13062-015-0054-9)
Supplement: Additional file 3: — Nearly complete virophage genomes from the gut metagenome. [file 13062_2015_54_MOESM3_ESM.pdf]

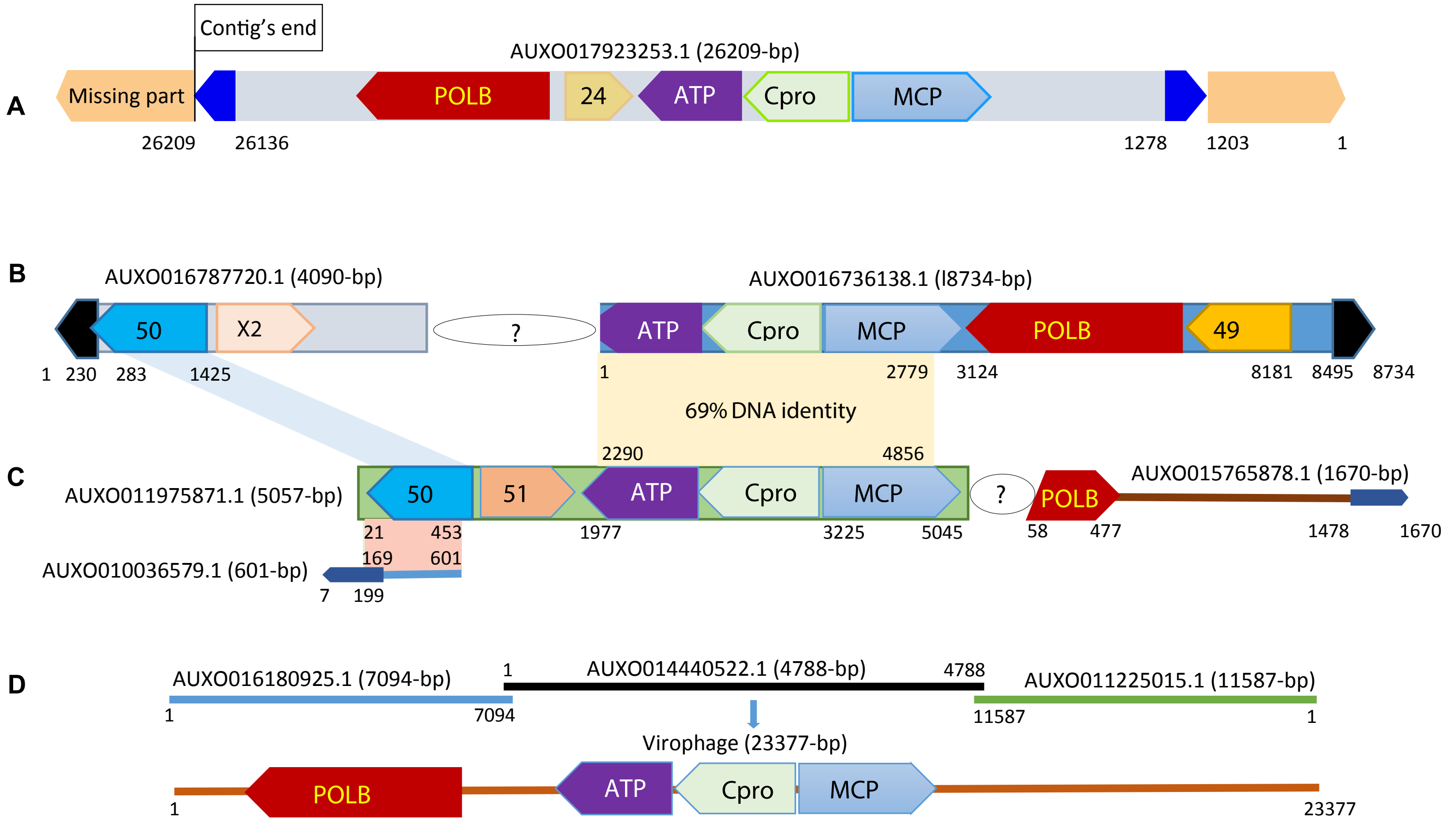

Additional file 3. Terminal inverted repeats and assemblies of gut virophages.
A: In the longest gut virophage identified in a single contig, blue arrows denote 97% identical 74-bp inverted repeats. Given that the 5' inverted repeat is at the contig terminus, it is most likely that the virophage TIR is much longer (brown arrows). In addition to the DNA polymerase B (POLB), ATPase (ATP), cysteine protease (Cpro), and major capsid (MCP) proteins, this virophage encodes 16 unclassified proteins that are not shown (excluding the "24" protein).
B: The second virophage was assembled from two contigs. The 94% identical 230-bp TIRs are marked by the black arrows. This virophage is incomplete, and its unknown internal part is marked by the oval. The conserved (ATP-Cpro-MCP)-coding DNA region is 69% identical to the homologous region in the third gut virophage (light yellow rectangle). The light blue area indicates the less conserved homologous region in these virophages coding for the unclassified "50" protein (30% protein identity; no significant DNA similarity). "X2" and "49" are unclassified proteins.
C: The incomplete third virophage was assembled from three contigs; the pink rectangle denotes overlapped 94% identical 5'- and 3'-terminal parts of AUXO011975871.1 (pos. 21-453) and AUXO010036579.1 (pos. 169-601). Two blue arrows indicate 99% identical 193-bp terminal inverted repeats of the assembled virophage (pos. 7-193 in AUXO010036579.1 and pos. 1478-1670 in AUXO015765878.1). The unknown internal part of the virophage is marked by the oval (it includes at its 3' terminus the missing N-terminal portion of POLB.
D: Partial assembly of the gut virophage from the overlapped AUXO014440522.1, AUXO016180925.1 and AUXO016180925.1 contigs. Other non-canonical proteins are not shown. The overlapped 100% identical regions are 40-bp long. The 5'- and 3'-terminal parts of the assembled 23377-bp virophage are not known.
